# Medication Reconciliation in the Surgical Setting: A Cross-Sectional Study in Polymedicated Patients

**DOI:** 10.3390/jcm15010270

**Published:** 2025-12-29

**Authors:** Mercedes Jiménez-Heredia, Vlada Zabrodotska-Maksymyuk, Carmen Carrión-Carrión, María Galiana-Sastre, Joaquin Ortega Serrano, Diego Cano-Blanquer

**Affiliations:** 1 Servicio de Farmacia, Hospital Clínic de Barcelona, Calle Villarroel 170, 08036 Barcelona, Spain; 2 Centro de Salud Miguel Servet, Departamento de Salud de Valencia-La Fe, Calle Amigos del Corpus S/N, 46025 Valencia, Spain; zabodotska_vla@gva.es; 3 Servicio de Farmacia, Hospital Clínico Universitario de Valencia, Avenida Blasco Ibañez 17, 46010 Valencia, Spain; carrion_carcar@gva.es (C.C.-C.); galiana_marsas@gva.es (M.G.-S.); 4 Departamento de Cirugía, Facultad de Medicina y Odontología, Universidad de Valencia, Avenida Blasco Ibañez 15, 46010 Valencia, Spain; joaquin.ortega@uv.es

**Keywords:** medication reconciliation, polymedicated patients, medication discrepancies, perioperative safety, pharmacist intervention

## Abstract

**Objectives**: This study aimed to assess the incidence, nature, and clinical relevance of medication discrepancies identified during the perioperative period in polymedicated surgical patients, and to examine factors associated with the occurrence of real discrepancies. **Methods**: A cross-sectional study was conducted in scheduled surgical patients admitted to the General Surgery department of a tertiary-care hospital. Eligible adults were required to be taking ≥4 chronic medications, have restored oral tolerance, and remain hospitalized for more than 48 h. Medication reconciliation was performed using hospital and primary care electronic records, complemented by a structured patient interview. Discrepancies were classified as justified or real according to SEFH criteria. Statistical analysis included descriptive methods, normality testing, correlation analyses, and generalized linear models. **Results**: Out of 270 assessed patients, 43 met inclusion criteria. A total of 282 medications were analyzed, with 243 (86%) showing discrepancies. 44% were real discrepancies, primarily due to unjustified omission. The average number of real discrepancies per patient was 5.7 (95% CI: 4.8–6.5). Cardiovascular (35.2%) and nervous system drugs (23.2%) were most affected. Real discrepancies with potential clinical severity accounted for 36.8%, including cases of asthma exacerbation, withdrawal syndromes, insomnia, and hypertensive crises. In 73% of pre-anesthesia reports, no specific recommendations regarding chronic medication management were provided. **Conclusions:** Medication reconciliation revealed frequent and clinically relevant discrepancies in this high-risk cohort of polymedicated surgical patients. Larger, more representative studies are needed to confirm these findings and to inform broader perioperative safety strategies.

## 1. Introduction

Advancements in surgical and anesthetic techniques have increased the number of elderly patients undergoing surgery [1], often with higher comorbidity, impaired renal/hepatic function, and complex chronic pharmacotherapy [2]. These factors are associated with lower survival and a higher incidence of adverse events [3].

During the perioperative period, most chronic treatments can be continued without additional risk; however, certain medications may amplify anesthesia- or surgery-related risks, and abrupt interruption may worsen underlying conditions or trigger withdrawal syndromes when surgical stress increases vulnerability [4].

One of the available tools to address this issue is medication reconciliation, defined as the formal and standardized process of obtaining a complete list of a patient’s prior medications, comparing it with the current hospital prescription, and analyzing and resolving any discrepancies identified [5]. The aim of the medication reconciliation process is to ensure that, throughout the entire continuum of healthcare, patients receive all necessary medications they were previously taking, prescribed with the correct dose, route, and frequency [6], and adapted to the patient’s clinical condition and the new hospital prescription. Medication reconciliation should be based on current evidence-based recommendations regarding the perioperative management of chronic medications [7,8,9]. Although these recommendations are rarely supported by randomized controlled trials, they are grounded in expert opinion, isolated clinical cases, or theoretical considerations extrapolated from experience with similar drugs. These recommendations serve as a guide to prevent omissions and/or unjustified delays once the patient regains oral tolerance to fluids and, consequently, the ability to resume their medication.

In this regard, the pharmacist’s role has been shown to be crucial in the medication reconciliation process. Numerous studies have demonstrated that pharmaceutical interventions can potentially reduce the occurrence of medication errors in perioperative settings, serving as a fundamental pillar for the successful implementation of medication reconciliation [10,11].

## 2. Materials and Methods

A cross-sectional study was conducted on patients undergoing scheduled surgery within the General Surgery department at a tertiary-level hospital. All surgical patients scheduled under the General Surgery service were selected based on the following inclusion criteria: patients aged 18 years or older, patients with polypharmacy (defined as taking more than three chronic medications), patients with oral intake reestablished after surgery or with tolerance to liquids at the time of the interview, patients with a hospital stay longer than 48 h, and those who had signed informed consent.

Exclusion criteria included: patients undergoing emergency surgery, patients with postoperative complications requiring extended stay in the Recovery Unit or with oral intake not yet reestablished, patients with cognitive impairment and/or without a caregiver.

The variables collected are presented in Table 1.

The variables were collected to obtain the most comprehensive pharmacotherapeutic history for each patient. Data collection followed a structured process: First, the hospital’s electronic prescribing systems were reviewed to gather information on medications administered during hospitalization, nursing instructions regarding oral tolerance, blood glucose monitoring, and other relevant clinical details. Second, primary care pharmacotherapeutic records were consulted to identify active chronic medications, documented allergies, and pertinent demographic information. Third, the electronic medical record was examined for additional variables of interest, including the pre-anesthesia report and surgical notes. Furthermore, a semi-structured interview was conducted with each patient and/or their caregiver by a nurse specifically trained for this task. Each interview lasted approximately 10–30 min and aimed to verify and supplement previously collected data, compiling the most complete list of chronic medications, including over-the-counter products (e.g., NSAIDs, antacids, vitamin and mineral supplements, herbal preparations). To ensure methodological rigor and minimize interviewer-related variability, all interviews were performed by the same trained nurse. Finally, discrepancies were analyzed and classified as either justified or real (see Table 2) by two independent pharmacists, in accordance with the 2009 SEFH consensus document on terminology and classification in medication reconciliation programs [5].

Data analysis was conducted using SPSS version 19, applying a 95% confidence interval and a significance threshold of *p* < 0.05. Qualitative variables were summarized using frequencies and percentages, while quantitative variables were described through means and confidence intervals, with normality assessed via the Shapiro–Wilk test and visual inspection. Event-based outcomes were analyzed using generalized linear models with a log link, evaluating model fit under a Poisson framework and considering a negative binomial approach when indicated. Multivariable models incorporated clinically relevant covariates, and results were expressed as incidence rate ratios with 95% confidence intervals.

## 3. Results

**Figure 1:**
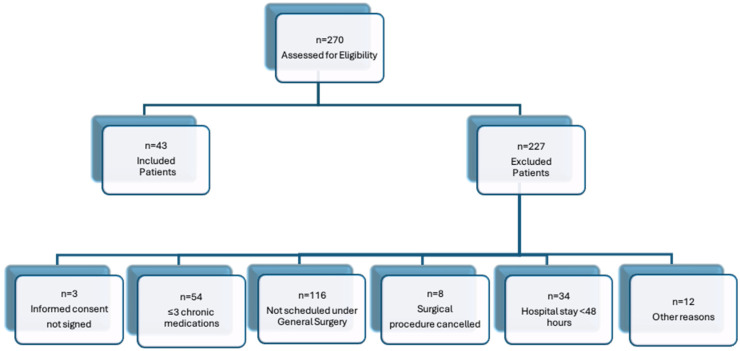
Patient Inclusion and Exclusion Flowchart.

A total of 43 patients were included from an initial pool of 270 eligible individuals who underwent scheduled surgery without complications at a tertiary-level hospital (Figure 1).

A total of 282 medications were recorded during the reconciliation process. The characteristics of the study population are presented in Table 3.

Regarding the general surgery procedures undergone by the included patients, the most frequent was bariatric surgery (23.3%), followed by hernia repair (20.9%), biliary surgery (11.6%), and colorectal surgery (11.6%).

The pre-anesthesia report was attached for nearly all patients (95.3%) and included recommendations regarding the management of chronic medication during the perioperative period. In 3.5% of cases, discontinuation of treatment was advised; in 20.2%, continuation of medication was recommended; and in 3.5%, substitution with an equivalent drug was suggested. However, in 73% of cases, no specific recommendations were provided in the pre-anesthesia report, despite patients being on chronic medication.

The average time to the onset of oral tolerance was 13.1 h post-surgery (95% CI: 11.1–15.0), although some patients tolerated oral intake as early as 6 h (11.6% of cases). Oral medication was reintroduced, on average, 6.3 h after the onset of oral tolerance (95% CI: 4.3–8.4).

When comparing the hospital prescription with the patient’s usual chronic medication and after conducting the interview, a total of 243 discrepancies were identified. Among these, the most frequent were justified discrepancies due to therapeutic substitution according to the hospital’s pharmacotherapeutic guidelines, accounting for 20% of cases (Table 4). On the other hand, 44% of the medications analyzed presented discrepancies classified as real, with omission of the prescribed medication being the most common.

A total of 93% of patients presented at least one real discrepancy, with an average of 43% of their chronic treatment affected by such discrepancies. No statistically significant differences were observed in the distribution of real discrepancies between sex-stratified groups (*p* = 0.684). The analysis identified a clear and statistically significant association between the number of chronic medications and the likelihood of real discrepancies. Both Pearson’s (r = 0.65; 95% CI: 0.44–0.80) and Spearman’s coefficients (ρ = 0.52; *p* < 0.001) demonstrated a moderate, positive correlation. In the adjusted Poisson model, medication count remained an independent predictor of discrepancies (IRR = 1.212; 95% CI: 1.141–1.288), corresponding to a 21% rise in expected discrepancies per added medication after controlling for age, sex, and presence of a caregiver.

Medications were classified according to the Anatomical Therapeutic Chemical (ATC) Classification System for evaluation. Among the ATC groups associated with real discrepancies, the cardiovascular system category (ATC group C) accounted for the highest proportion (35.2%), with lipid-lowering agents (C10) being the most prevalent subgroup (19.2%). This was followed by the nervous system category (ATC group N), which represented 23.2% of discrepancies, with antidepressants (N06) being the most frequently involved subgroup (9.6%).

**Figure 2:**
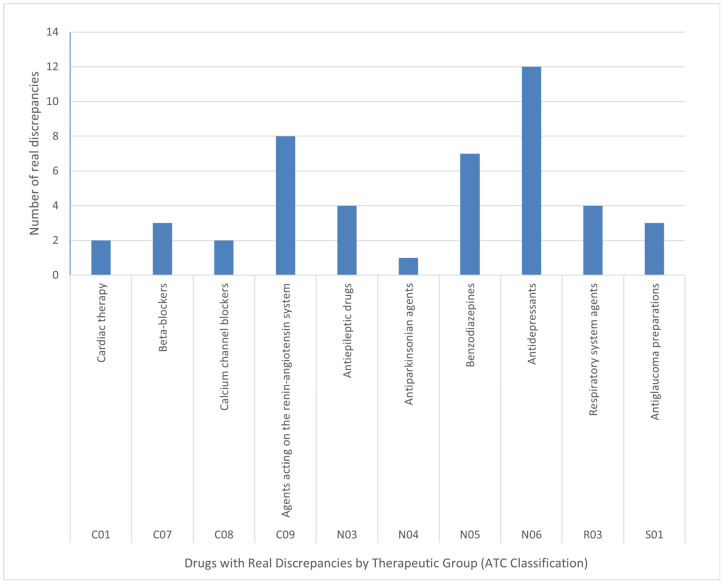
Drugs Involved in Potentially Severe Real Discrepancies.

Regarding the assessment of the severity of identified real discrepancies, the potentially most serious cases—those involving medications that should not be discontinued due to the high risk of acute withdrawal syndromes, clinically relevant adverse effects, and/or postoperative complications—accounted for 36.8% of real discrepancies. These cases predominantly involved drugs acting on the cardiovascular system (e.g., antiarrhythmics, antihypertensives) and the nervous system (e.g., antidepressants) (Figure 2).

Among the most clinically relevant events identified was an asthma exacerbation secondary to the omission of the patient’s usual inhalation therapy, which led to bronchospasm in a patient with compromised pulmonary function following abdominal surgery. This required urgent treatment with aerosolized medications.

Another case involved a withdrawal syndrome resulting from the interruption of chronic treatment with opioids, anxiolytics, and antidepressants (tricyclics, SSRIs, and SNRIs), which triggered symptoms such as anxiety, insomnia, and abdominal cramps. The patient required rescue opioid doses following the event.

Additionally, a case of persistent insomnia was observed following the withdrawal of anxiolytics, and a hypertensive crisis (systolic blood pressure > 140 mmHg) occurred after the suspension of antihypertensive therapy, requiring rescue treatment with sublingual captopril and clinical monitoring.

## 4. Discussion

Surgical patients are particularly vulnerable to risks and complications arising from the withdrawal of chronic medications [12]. If we add to this fact that more than half of the patients who undergo surgery are usually elderly, with chronic pathologies and polypharmacy, this risk of suffering conciliation errors may be increased [13]. In addition, the risk of reconciliation errors increases proportionally with the duration of treatment interruption. Therefore, it is essential to include these patients in medication reconciliation programs to optimize clinical safety throughout the care process.

The findings of this study indicate the presence of a high incidence of real discrepancies within this cohort of surgical patients with polypharmacy. Of the 282 medications analyzed, 86% exhibited some type of discrepancy, with 44% classified as real discrepancies and an average of 5.7 real discrepancies per patient. These results are consistent with previous evidence showing that more than half of surgical patients experience unjustified differences between their usual medication regimens and in-hospital prescriptions [14,15]. Unjustified omission was the most frequent discrepancy (43%), aligning with published data [16,17] highlighting a significant clinical risk for polymedicated patients with chronic diseases. Furthermore, the study confirms that the number of medications is a strong and consistent predictor of real discrepancies.

In the analysis of the severity of identified real discrepancies, a greater involvement was observed among therapeutic groups corresponding to antidepressants (ATC group N06), followed by medications acting on the renin–angiotensin system (ATC group C09). This finding is consistent with various published guidelines [7,8,9,18] that identify these groups as critical in perioperative management due to their impact on the patient’s clinical stability. Although this study was not specifically designed to detect complications directly attributable to the withdrawal of chronic medications, the four clinical cases observed show similarities with those described in previous research [4,16].

Regarding the reintroduction of medication following surgery, in our study this occurred, on average, 6.3 h after the onset of oral tolerance. Oral tolerance was achieved at a mean of 13.1 h post-surgery. This interval exceeds the recommendations established in clinical guidelines [19], which advocate for earlier reintroduction of nutrition—and consequently oral medication—whenever the patient’s clinical condition permits.

With respect to pre-anesthesia reports, a significant lack of specific guidance regarding the management of chronic medication was identified. In 73% of cases, no concrete recommendations were provided. Although these reports adequately address the withdrawal of drugs that may interfere with the surgical procedure, they do not offer equally precise guidance on the need to maintain or reintroduce chronic medication after surgery.

One of the main limitations of this study is its cross-sectional design, which restricts the assessment of delays or omissions in the administration of chronic medications to those identified at the time of the interview. The relatively small sample size further limits the strength and external validity of the findings. Additionally, a methodological limitation arises from the exclusion of cases in which the attending physician modified the prescription based on pharmacist recommendations following appropriate validation prior to the research team’s review, as these instances were not classified as real discrepancies. Nonetheless, in all instances where a real discrepancy with potential clinical relevance was detected, immediate notification was provided to the responsible healthcare professional to ensure prompt resolution.

In conclusion, our findings suggest that polymedicated surgical patients may be at risk of postoperative complications related to the omission of chronic medications during the perioperative period, as observed in our cohort. This underscores the importance of expanding pre-anesthesia reports to include explicit recommendations for postoperative pharmacological management. Moreover, the results highlight the essential role of pharmacists in the medication reconciliation process, together with the contribution of nursing staff, whose active involvement supports early identification of oral tolerance and timely reintroduction of chronic therapies. Integrating these actions within a framework of interdisciplinary communication and standardized clinical practices is crucial to enhance patient safety throughout the continuum of care. However, larger and more representative studies are needed to confirm these observations and to determine their broader applicability.

## Figures and Tables

**Table 1:** Variables Collected.

Variable	Description
Interview date	Day and month
Date of birth	Month and year
Sex	Male or Female
Known allergies and intolerances to medication	Specified by patient or caregiver
Date of surgical intervention	Approximate time of entry and exit from operating room
Date of initiation of oral medication and/or liquid tolerance	As exact as possible
Primary diagnosis	Reason for scheduled surgery
Type of surgical intervention	Procedure performed
Patient accompanied or not	Presence of caregiver or companion
Chronic medication	Active ingredient, dose, frequency, route of administration, date and time of reinitiation post-surgery, hospital electronic prescription (Yes/No), primary care electronic prescription (Yes/No), brought from home (Yes/No), without medical prescription (Yes/No)
Pre-anesthesia report	Medications to be discontinued, maintained, or substituted, and duration
Type of discrepancy	Justified discrepancy or real discrepancy
ATC code	Anatomical Therapeutic Chemical Classification System

**Table 2:** Types of Discrepancies.

Code	Type of Discrepancies	Description
1.	Justified discrepancy	
1.1		Omission of a chronic medication, or change in dose, frequency, or route of administration based on the patient’s clinical condition
1.2		Therapeutic substitution according to the hospital’s pharmacotherapeutic guidelines
1.3		The patient brings and takes their chronic medication by medical order (anesthesiologist and/or surgeon), without it being prescribed in the hospital’s electronic prescription system
2.	Real discrepancy	
2.1.		Omission of a necessary chronic medication: not prescribed in the hospital’s electronic system and not provided by the patient, or provided but the patient is unaware they should take it
2.2.		Duplication of necessary chronic medication: prescribed in the hospital’s electronic system and also brought and taken by the patient (same active ingredient or same therapeutic group)
2.3.		Initiation of chronic medication in the hospital’s electronic system that the patient was not previously taking, without clinical justification
2.4.		Different dose, route, or frequency of chronic medication in the hospital’s electronic system compared to how the patient was taking it, without clinical justification

**Table 3:** Characteristics of the study population.

Sex	Female (n, %) Male (n, %)	23 20	53.5% 46.5%
Caregiver	Yes (n, %) No (n, %)	41 2	95.3% 4.7%
Age (Median, Range)		66 years	36–89 years
Medications per patient (Median, Range)		6	4–14
Length of hospital stay (Mean, 95% CI)		6.8 days	5.3–9.8

**Table 4:** Distribution of Medications According to the Reconciliation Process and Type of Discrepancies.

	Total Medications (=282)	
	*n*	%
**Without discrepancies**	39	14%
**With discrepancies**	243	86%
**Justified discrepancies**	118	42%
*Omission or modification of dose, route, regimen, or frequency based on clinical condition*	*37*	*13%*
*Therapeutic substitution according to hospital formulary (GFT)*	*55*	*20%*
*Medication taken by medical order (anesthesiologist and/or surgeon) without being recorded in the hospital electronic prescription system*	*26*	*9%*
**Real discrepancies**	125	44%
*Omission of necessary chronic medication*	*122*	*43%*
*Modification of dose, route, or frequency without clinical justification*	*0*	*0*
*Initiation of unnecessary chronic medication*	*0*	*0*
*Duplication of chronic medication (same active ingredient or therapeutic group)*	*3*	*1%*
**Average number of real discrepancies per patient** (95% CI)	5.7	4.8–6.5

## Data Availability

The original contributions presented in this study are included in the article. Further inquiries can be directed to the corresponding authors.
